# Re-evaluating the structure of consciousness through the symintentry hypothesis

**DOI:** 10.3389/fpsyg.2023.1005139

**Published:** 2023-11-21

**Authors:** David Rail, Andrew Selby

**Affiliations:** ^1^Consultant Neurologist, Sydney, NSW, Australia; ^2^Independent Practitioner, Kings Langley, United Kingdom; ^3^AECOM, St Albans, United Kingdom

**Keywords:** projective consciousness model, phenomenal self, self-identity, PMIR, transcendental structuralism, symmetry, intentionality, PCM

## Abstract

The Projective Consciousness Model and its extension to the phenomenal selfhood model are the generic invariant structures of consciousness through five symmetries. They include the following: situated 3D spatiality; temporal integration through encompassing the three “nows” that constitute the Now; multimodal synchronic integration; relational phenomenal intentionality; and consciousness entails a pre-reflective awareness of the uniqueness of the phenomenal self. These symmetries stem from the evolution and emergence of the phenomenal self through modeling, and that is realized through the phenomenal modeling of the intentionality relationship. We propose that this set of symmetries is based on and can be explicated in terms of a more fundamental symmetry, symmetry-based modeling. The proposal stems from (a) Kant's transcendental structuralism, which asserts that “Objects” conform to models prescribed through the inherent structure of the phenomenal mind, and (b) Cassirer's proposition that a mathematical group underpins this structure. To validate our proposal, we stipulate this group defining symmetry-based modeling and its emergence and adaptation into structuring the Now. We find that Cassirer's group requires a more powerful dual quaternion operator to be able to support intentionality and the five symmetries. We propose that the efficacy of this operator stems from unifying symmetry-based modeling and intentionality as “symintentry.” Symintentry, we argue, is not just a new form of symmetry but is the archetypical form of symmetry. Symintentry provides fresh insights into the nature of symmetry, intentionality, and consciousness.

## Introduction

Everything has structure—even consciousness, which is arguably the most elusive, ephemeral, and indefinable process. The Projective Consciousness Model (PCM) (Rudrauf et al., 2017), through its extension to the phenomenal selfhood (Williford et al., 2018), models the generic and phenomenological invariant structures and functions of consciousness. The model provides a dual phenomenological and functional (cybernetic) role that consciousness plays for the embodied organism. The PCM provides a comprehensive theory of the structural basis for the emergence of phenomenal selfhood (PS) through self-modeling (PSM). In consciousness, a consciously experienced self and a first-person perspective emerge in the human information-processing system (Metzinger, 2004, 2007a). Through PSM, operations simulate and emulate properties of their own information processing.

In PCM, the structure of consciousness is conceived in terms of five invariants or symmetries (St5) (the authors of PCM have indicated the basis for the multidisciplinary literature on each of these “invariants.” There is phenomenological and psychological evidence for each. Following are some of the key references for the invariants indicated: Castoriadis, 1980; Husserl, 1983; Rudrauf et al., 2003; Metzinger, 2004; Tononi, 2004; Baars, 2007; Williford et al., 2013; Oizumi et al., 2014; and Rudrauf et al., 2017).

First, situated 3D spatiality: PS is central to a 3-dimensional perspectival view of the world that links perception, imagination, and planning. Second, PSM is supported through temporal integration encompassing the three “nows” that constitute the Now. These nows are the primal impression, retention, and protention (i.e., the immediate present, past, and future; Vrobel, 2007). Third is multimodal synchronic integration, in which a range of qualitative and representational components synchronize to function as a unified whole (Now). The fourth invariant is relational phenomenal intentionality (PMIR). PMIR provides a functionalist model of the system in the act of experiencing the subject-object relationship (Metzinger, 2004, 2007a). When experiencing, we not only represent objects or states, but we also co-represent the representational relationship itself. Co-representation realizes phenomenal experiences as subjective and perspectival. The fifth invariant is that consciousness entails a pre-reflective awareness of the uniqueness of PS.

The structure of consciousness (St5) depends on the co-evolution and emergence of the “virtual organs” PSM and PMIR (Metzinger, 2007a,b). It is important to determine whether the five invariants are the ultimate grounds for consciousness. In that regard, we contend that St5 is based on and can be explicated in terms of a more fundamental symmetry—symmetry-based modeling (SyBM). First, PMIR implicates all the other aspects of St5 (Williford et al., 2018). Second, the development of PSM and PMIR depends on modeling. Furthermore, all biological processes are inferential from evolution (the genome) through to conscious processing, i.e., the Now^1^ (Friston, 2018). Consciousness as a biological process develops through the evolution and emergence of inference and modeling.

We see that the role of SyBM in structuring consciousness can be further elucidated by revisiting Cassirer's response to Kant's transcendental structuralist thesis (Kant et al., 1996; Kauark-Leite and Neves, 2016). Kant revolutionized the understanding of the relation between percepts and external objects by shifting the focus of epistemology from the structure of the external world to the structure of the mind. The Copernican Revolution implied that our perceptions and thoughts conform to models prescribed by the inherent structure of the phenomenal mind. In that regard, our primary cognitive activities depend on making, validating, and applying conceptual models (Hestenes, 2015). According to Kant, “The fundamental laws of nature, like truths of mathematics, are knowable precisely because they do not describe the world as it really is, but rather they prescribe the structure of the world as we experience it” (Hestenes, 2015).

Kant's seminal idea has continuing relevance for our understanding of top-down modeling in determining the structure of the mind. Swanson (2016) showed that Kant anticipated several core aspects of the predictive processing (PP) paradigm^2^ concerning the generation of top-down models. PP explains how brains are able to track real-world causes using only sensory effects (Körding et al., 2007). While PP was concerned with learned priors, Kant focused on explaining *a priori* features of cognition and perception. For Kant, *a priori* is what Friedman (2000) referred to as a relativized and dynamical conception of mathematical-physical principles that underpin structure.

Swanson's support of Kant was based on the following five principles:

(a) “Objects” conform to our cognition, i.e., our hypotheses.
(b) Models or transcendental schema function through a causal matrix to employ endogenously generated rules modeling input patterns (Clark, 2015). Schemata bridge the gap between concepts and perceived “objects.”
(c) Space and time are endogenously generated internal structures that constrain our perception of “objects.”
(d) Through a process called “analysis-by-synthesis,” we model incoming sensations by comparing them to internally generated (synthesized) patterns (Hatfield, 2006).
(e) Imagination is key to the synthetic function of the generative models in facilitating perceptions.

Subsequently, Cassirer supported Kant's transcendental structuralist thesis by claiming that a mathematical group (Cassirer's group, CG) was the basis for our cognition and perception in self-object relationships^3^ (Cassirer, 1944; French, 2001; Lovrenov, 2006; Kauark-Leite and Neves, 2016; Biagioli, 2020). That claim is important because, through fully stipulating CG for the first time, we can elucidate the role of symmetry-based modeling in phenomenal experience-making, validating, and applying conceptual models. Furthermore, we can explicate how biological processes evolve and how biosemiotic processes emerge to conscious processing in the Now. CG can explicate Kant's transcendental structuralist thesis by actively linking the demands of the knowing subject and the definition of its object (Bitbol et al., 2009). The group enables us to define SyBM and validate our proposal that underpins St5.

## Our approach to modeling the development of modeling

We explore the intersection of neurophenomenology and transcendental structuralism to determine the *a priori* structure of (the preconditions for) phenomenal experience and how that facilitates and enriches lived experiences (Khachouf et al., 2013; Hunt and Schooler, 2019).

To validate our proposal, we engage in a thought experiment to study the development of SyBM and show how that process can realize St5. We conceive development in terms of AEEN—adaptation through evolution and emergence in Now (conscious processing). We propose the AEEN SyBM hypothesis (ASH) that AEEN SyBM of self-object relationships provides the foundation for St5. We validate ASH by determining how AEEN SyBM realizes St5 from two perspectives.^4^ In Section 1, we focus on the emergence of CG to generate Now. In Section 2, we consider the influence of adaptation on the development of the structure of Now.

Section 1: We fully stipulate CG as the basis for the SyBM of epistemic relationships. To be able to specify the emergence of CG into the Now as the basis for St5, we find that CG cannot support intentionality (PMIR) and validate ASH. We propose that to support intentionality, CG must have evolved through a mutation leading to a more powerful operator (dual quaternion (DQ) rather than just quaternion). Subsequently, through evolution, SyBM emerged as the duality—SyBM-intentionality to function as “symintentry-based modeling” (we abridge this as “symintentry”). We propose that symintentry was the evolutionary step that was necessary for the emergence of the structure of consciousness. Furthermore, symintentry resolves symmetry breaking in the mind ~ brain functioning at metastability^5^ (Kelso and Tognoli, 2009).

**Figure 1:**
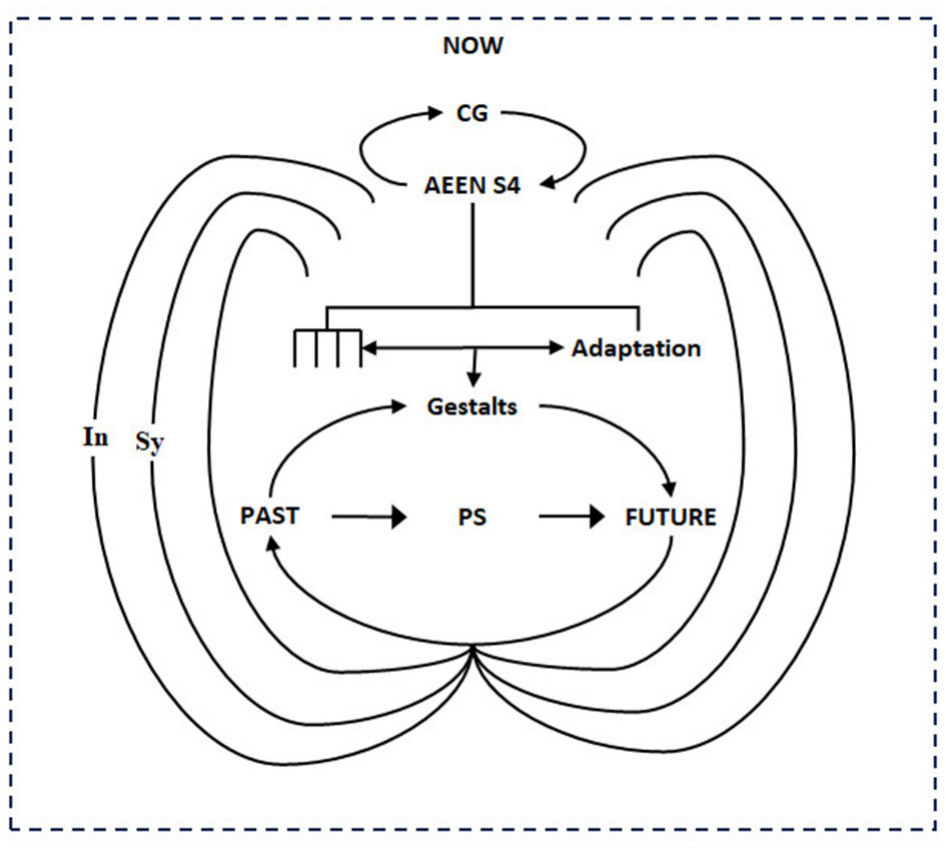
How the a priori structure or preconditions for phenomenal experience emerge in ontogeny. The figure depicts the adaptive emergence (AEEN) of CG to S4 [the top loop] to CG* (the bottom loop) to generate gestalts. The gestalts realize the transformation of the phenomenal self (future reconciled with past) through phenomenal self-modeling and symintentry. In more detail, the adaptive emergence of CG to S4 (Sy5, Sy6) [depicted as four “prongs”] and adaptation leads to a higher-level group CG*. CG* is a highly embedded four-level group structure representing transcendental-semiotic-epistemic-ontic “levels” of phenomenal experience. The symintentry operator enables autocatalytic and cyclical integration of the four levels by forming an Ouroboros loop [depicted as the 3-loop structure]. The Ouroboros loop function involves (inner level) self-referential presentation of self to “other.” The second level is representation [symbolic (Sy)]. The third level is representation of representation through intentionality (In). SyTO “tunes” this extensive “vertical” fractal structure, enabling the self to potentially become any or all “other” through dynamic gestalt formulation in real-time, i.e., Now. The highly efficient Ouroboros loop structure supports creativity and imagination.

We are now in a position to stipulate how AEEN symintentry (symintentry-based modeling) can generate St5 (see Figure 1 for the outline). To do that, we propose that S4, the four facets of symmetry, i.e., SyBM (Mouchet, 2013), constitutes the structure of SyBM. That is, S4 realizes St5 through AEEN. More specifically, S4 involves symmetry breaking (in each Now), and that process is the basis for AEEN. We indicate the following key factors that determine AEEN CG to S4 in Now:

(a) Five aspects of self-organization that determine emergence in Now.
(b) The properties gained from DQ.
(c) The six modes of function of SyBM (Sy6)^6^ (Mouchet, 2013).
(d) The properties of SyBM gained from adaptation through Shaw's ecological model of intentionality (Shaw, 2001).

In Section 2, we consider the influence of adaptation on the development of S4 by showing that AEEN symintentry is ideal for encompassing Shaw's ecological model of intentionality (Shaw, 2001). The symintentry operator (SyTO) functions as the control parameter, enslaving extensive complementarities to formulate optimal goal paths in Now. To fully reconcile the role of AEEN S4 in structuring Now, we propose that CG emerges into a higher-level group called CG^*^ that encompasses semiotic self ~ other. In CG^*^ classification, the group-based levels of description (LODs) of fractal space-time (List, 2019) emerge as four levels: transcendental, semiotic, epistemic, and ontic. SyTO, as the transcendental operator, realizes the integration of the four levels as Ouroboros loops (Thomsen, 2011, 2015): first, self-referential presentation of Self to Self as other. The second is symbolic representation. Third, intentional (symintentry) representation of representation.

We show that ASH is resolved through the correspondence between AEEN S4 emergence to CG^*^ and the structure of consciousness (St5). We argue that the emergence of S4 to CG^*^ advances and refines PCM.

## Cassirer's group rationales

Before we stipulate CG, we provide further background concerning the rationales for Cassirer's group. Cassirer was percipient in realizing the role of group symmetry as a general purpose mechanism in modeling epistemic relationships (French, 2001; Lovrenov, 2006). Cassirer realized that the object of perceptual experience is not a thing *or hic et nunc*. Rather, there were non-physical elements, i.e., structural elements, involved in the perception (Cassirer, 1944) that make perceptual judgments possible. The structural elements were based on mathematical groups, each of which had an ideal formal structure and obeyed strict mathematical rules (Friedman, 2003; Heis, 2007).

The symmetry principles are represented group-theoretically so the relevant group (in particular CG) lays down the general conditions in terms of which something can be viewed as an object (French, 2001). Cassirer argued that group theory does not represent reality but is an instrument endowed with transcendental function. That is, the group provides the active link^7^ between the demands of the knowing subject and the definition of its object (Bitbol et al., 2009; Bitbol and Osnaghi, 2016). The central role of CG and SyBM is unrealized because this group remains undefined, let alone correlated with neurological functioning. By defining CG, we can characterize SyBM and how that advances transcendental structuralism as the basis for the structure of consciousness, i.e., St5.

Our thesis advances the role of SyBM from three perspectives. First, Cassirer's approach to SyBM was founded on Felix Klein's Erlangen Program (Cassirer, 1944; Ihmig, 1999). Klein realized that the many different types of geometry (existing at that time, circa 1870) could be integrated by adopting a new direction of thought (Klein, 1893). The general properties of figures are characterized by the fact that they can be mapped from one particular figure to another one in terms of a suitable continuous transformation (Schiemer, 2018). Klein's theory defined the powerful role of invariants over specific transformations in describing the global nature of spatial organizations (Galati et al., 2010). Klein's ideas enabled a reclassification of geometries in terms of a structure based on a hierarchy of group-based symmetry transformations.

Klein's important insight was that certain classes of spatial transformations, equipped with a suitable composition function, form a group in the algebraic sense of the term that, in a way, encodes the abstract content of a given geometry (Schiemer, 2018). Every geometry, in its general concept and aim, is a theory of invariants with respect to a certain group, and the special nature of each depends upon the choice of this group. Cassirer developed the analogy between the kinds of structures that gain formal objectivity in geometry (as objects of knowledge) and those that characterize empirical experience (Bundgaard, 2002).

The second role of SyBM was that Cassirer conceived it as key to modeling the epistemic function of Kant's schemata. In particular, SyBM provides a new way to express the conservation laws for dynamical systems that underpin physics and, in particular, Bohr's view of quantum mechanics [see Brock (2009) for the following analysis]. Bohr considered complementarity to be a generalization of the Kantian ideal of causality. Causality is a principle of knowledge and not an ontological claim. The schemata translate a temporal series of spatial manifestations into a description of the characteristic kinematics and dynamics of the physical objects posited in theoretical thought. The Kantian idea of schematism is key to an understanding of establishing “mechanics” in relation to a field of physical experience. In effect, space-time coordination and conservation laws for dynamical systems are complementary aspects of causality. Subsequently, Cassirer realized that CG enabled a reformulation of these physical laws and principles (such as the least action principle) that are constituted in and govern our a priori knowledge of the world.

By determining CG, we can show that SyBM is the most fundamental symmetry because it underpins St5. Furthermore, SyBM enables us to reformulate the laws and principles that have developed through phyloontogeny and the emergence of Now, which epitomizes the function of consciousness.

The third role of SyBM follows from Cassirer's realization that group theory can also model semiotic relationships. The semiotic function of CG is essential for SyBM to underpin consciousness. Therefore, we conjecture that the epistemic function of CG must be expanded through the emergence of intentionality. That is, human intentionality emerges from SyBM through a process we call “symintentry.”

Cassirer considered that group theory could not only structure self-object relationships but is applicable to all human knowledge (Cassirer, 1944). Furthermore, through symbolic prägnanz and the theory of perception, group theory could be generalized to account for semiosis (Cassirer, 1957; Leroux, 2011; Parszutowicz, 2015).

## Section 1: AEEN SyBM: the emergence of CG to generate Now

### We stipulate CG as the basis for SyBM

To determine AEEN symintentry, we need to define CG as the basis for SyBM of phenomenal self-object relationships. Chester (2002)^8^ proposed a group that epitomizes Cassirer's views on the group-symmetry basis for knowing “objects.” Chester contended that group theory constitutes the object of scientific inquiry because groups codify the axioms of scientific enterprise.

We know the nature of entities in the physical world by realizing that the “object” is part of a system. The object is perceived and becomes known through integrating measurements from a range of perspectives or coordinate systems. Each altered scrutiny or transformation of the individual's coordinates registers different views of the same object. The correlation of the set of all views, perspectives, or altered scrutinies establishes the object of scrutiny.

Altered scrutiny is an observer's construct that reweaves the fabric of descriptive space as the observer moves to another frame of reference to record measurements. Altered scrutiny is the action that puts the observer in various positions to make new measurements. Any change in the reference frame depends on the rotation of the observer-based coordinate system.

The set of altered scrutinies conforms to the definition of a group under the composition of rotation. First, the law of composition is just sequential action. Second, the set is closed; from a state of the system, an altered scrutiny can only produce another state of the same system. Third, the elements are associative because altered scrutinies are just a series of sequential actions. Fourth, the system has natural inverses, i.e., undo the alteration in scrutiny. Finally, the identity element is satisfied by doing nothing, i.e., no change.

Chester proposes that the group logic formalizes how science and the individual can identify entities in the physical world in a consistent and repeatable manner. He claims that symmetry coincides with identification and identity.

### Quaternions are the operator for CG

Chester stipulated the general structure of the group that characterizes modeling of altered scrutinies of rotation but did not specify it, except to say that it is a Lie group and that in real 3D space, it would be SO(3). We now further specify this group. We define its operator as quaternion because the change in the reference frame depends on the rotation of the observer-based coordinate systems.

Quaternions are a non-commutative algebra widely used in computer animation and satellite tracking (Pletinckx, 1989; Jia, 2022). Unit quaternions can generate altered scrutiny, i.e., enabling all 3D rotations via the double rotation operator (also known as the “sandwich function;” see Appendix 2 for more details). Unit quaternions are a member of the 3D rotation group SO(3), a Lie group that provides an efficient and robust mathematical process for dealing with invariant rotation of objects in three-dimensional space. Euler angles and other trigonometric functions do not have to be formulated relative to the Cartesian coordinates. Importantly, quaternion rotations do not suffer from “gimbal lock,” the effect of a mathematical singularity that occurs in other methods when rotating the coordinates to certain positions. Furthermore, quaternion rotation in 3D space provides a rigid body rotation that keeps an object's form invariant and allows for the shortest path (geodesic) interpolation (Kuipers, 1999).

### The coordinate transformation network provides the elements of CG

We have seen in Chester's group that the object is perceived and becomes known through integrating measurements by rotating through a range of coordinate systems. We consider the elements of CG to be the “altered scrutinies” or “coordinate transformations” founded on the coordinate transformation network (Cohen and Andersen, 2002).

Research on gain field encoding shows that gain-modulated neurons generate coordinate transformations (Andersen, 1997; Salinas and Sejnowski, 2001), integrating egocentric and allocentric coordinate systems. The framework of the Erlangen program has had a major influence on research in perceptual psychology (Galati et al., 2010). Recognition and categorization depend on the amount of geometrical transformation for most transformation groups of the Erlangen program (Graf, 2010). However, the concept of object-based transformations must change to perceptually based coordinate transformations through analog coordinate transformations. The coordinate transformation network is a process-based and dynamic geometrical framework that aligns input and memory representations using analog coordinate transformations so that the different levels can correspond (Graf, 2010). The input from gain-modulated neurons is taken multiplicatively to form the gain field.

### Properties gained from the DQ operator

To this stage, we find that from a developmental perspective, AEEN CG underpins epistemic relationships as in self-object identification through a form of group homomorphism, while it underpins spatial relationships in terms of the Erlangen program. We contend that CG cannot underpin the development of intentionality as in the representation of representation (PMIR) and semiotic self-other relationships. CG needs to have gained a more powerful DQ operator through the evolution and emergence of SyBM to function as a “symintentry.”

To support our argument, we outline the properties CG could gain through the DQ operator (also see the precis of the mathematical function of DQ in Appendixes 2, 3). First, 3D spatiotemporal transformations. The unit DQ is a Clifford sub-algebra [a Lie group SE(3) of which the 3D rotation group SO(3) is a sub-group] that can efficiently and robustly model Euclidean transformation in rotations, translations, scaling, reflection, and inversion. The DQ has a mathematical structure that allows the mapping of 3D spatial information onto the three dual vector components. By introducing an additional mathematical process (See Appendix 2), the DQ can incorporate a sequential count in the normally unoccupied dual scalar component. We posit that this dimensionless number acts as a time-base. Furthermore, with this proposed process, the DQ mathematics will allow different values (relative to the current Now) of the time base to be inserted into the dual scalar, hence enabling the conception of past or future events, as well as current ones. We contend that this facility is an essential feature of any such mathematical model.

Second, DQ provides a number system and algebra that support a group capable of capturing and transforming dynamic information in a Galilean space-time form (Ozyesil et al., 2018). The DQ can generate Cartan frames relative to one's perceptual frame of reference (Colón, 2015). These frames are rotated by a stream of unit quaternions that facilitate the capture of spatial events against a time base. The frames become the analogs of temporal order for integrating multiple coordinate systems that are necessary for task resolution. Cartan moving frames can integrate multi-level symmetries that are necessary for planning movements (Bennequin et al., 2009).

Third, DQ transformations model rigid body transformations and embodied tasks such as reaching and grasping (Schilling, 2011). We propose that this property enables the modeling of embodiment. Therefore, DQ operations reduce complexity.

Fourth, the ε operator is nilpotent, which has the counterintuitive property that ε is not equal to zero, and yet ε^2^ (and all higher powers) does equal zero. The multiplication of two or more unit DQs (or their conjugates) is converted into another unit DQ in which the four dual components are added together. This linearizes global function.

Furthermore, with respect to the simplifying capacity of the DQ operations, these can directly apply the symmetry operations of a combined body rotation followed by translation (or vice versa) in 3D space. Rotation calculations can be carried out without the use of matrices or Euler angles in a very efficient manner (i.e., minimal calculation steps) and, importantly, without incurring the singularity called “gimbal lock” that some other methods are prone to under certain conditions. The operation of translation is equally robust and is greatly simplified mathematically due to the properties of the nilpotent dual number (ε).

Fifth, we contend that under AEEN, the DQ operator in CG can account for intentionality. That further supports our proposal that intentionality can derive from symmetry (SyBM). The screw function of DQ provides a “vector” or “aboutness” quality to operations. DQ facilitates perception of the self-in-action in goal seeking. The perception-action cycle (PAC) and symbol grounding have also been modeled in terms of DQ (Leclercq et al., 2013; Bayro-Corrochano, 2020).

We find that DQ operations lead to a “symmetry-breaking” process that could realize intentionality. This is attained by resetting selected parts of the conceptual model(s) generated by symintentry. Resetting is achieved by raising the DQ to the power of zero (see Appendix 5). This nullifies all the dual components of the DQ (representing space and time), leaving just the scalar value of 1 (the identity) in a DQ form. The focus of the intentional state is thus reduced to zero. Effectively, the DQ components are zeroed and cease to exist, but they can, if required, be reinstated from memory. The process resets the symmetry in the DQ to a neutral setting (akin to but different from “breaking symmetry”). A new symmetry can be formed to complete the cycle. This is implemented by updating symintentry by generating conceptions or perceptions and then “writing” these onto the blank DQ. The DQ operations hold a “snapshot” frame in fractal memory.

Sixth, DQ can underpin the development of a sense of “space” in terms of projective geometry^9^ (Gunn, 2019) that we conceive as the basis for 3D situated spatiality, in particular as proposed in the PCM models (Williford et al., 2018). In PCM, it is proposed that 3D projective space (which may or may not include metrics) is necessary for the generation of consciousness. The authors also propose that the projective linear group [PGL(4)] is the mathematical system required to achieve this, and they cite the use of 4 × 4 matrices applied to homogeneous coordinates. It turns out that the mathematical properties of unit DQs can simply and directly generate the scaling operations necessary for a projective geometry based on a distant spherical “screen,” i.e., the retina. The inverted received image, via the mathematics of the unit DQ, has a very simple transformation for re-inversion, and it is proposed that the brain then uses unit DQs to generate the “lived space” illustrated in Figure 4 in Williford et al. (2018). As a result of the projective geometry, the self (PS) becomes central to transformations that encompass the “other” (see Appendix 3 for more details).

Seventh, DQ enables transformations that map the dynamics of self to others (in the manner of local to allocentric transformation described by Bremner and Andersen, 2012). In other words, DQ-based transformations can simulate the operations involved in gain fields in multimodal integration in the posterior parietal cortex (Andersen, 1997). The gain field accounts for the integration of self (intrinsic coordinates) and object or “other” (extrinsic coordinates).

Finally, eighth, the iteration of the DQ generates fractal time that we propose functions as the manifold that supports the structure and function of S4 in Now.

### The derivation of the structure and function of Now

To reiterate, ASH proposes that AEEN symintentry of self-other relationships (AEEN symintentry) provides the foundation for St5. We propose that CG, along with the DQ operator, can support intentionality. We now show how CG emerges through AEEN symintentry to generate the structure-function of Now (phenomenal experience). We propose that AEEN S4 resolves ASH—the hypothesis that AEEN SyTBM underpins the structure of consciousness (St5). AEEN SyTBM, or self-organization and adaptation, is conceived in terms of the following entries:

(a) The components of self-organization (see Appendix 1).
(b) The properties gained from the DQ operator (outlined previously).
(c) The six modes of function of SyBM (Sy6; Mouchet, 2013). Symintentry classifies, builds representations, constrains, and unifies interactions in models, extracts universal properties, bridges integrable and chaotic dynamics, and predicts selection rules.
(d) The properties of intentionality gained from adaptation, as conceived by Shaw (2001).

We now consider the contribution of each aspect of S4 in turn and how they integrate to form the structure-function of Now and St5. In Table 1, we compare CG and the structure of Now in terms of the four facets of symmetry, i.e., S4.

**Table 1:** The correspondences realized through AEEN that correlate CG (SyBM) and the structure of Now (S4).

**Aspects of function at each level of mapping of AEEN**	**Cassirer's Group**	**Now in terms of S4**
Transformation operator	Quaternion rotation and DQ translation and scaling	SyTO functions through 5 symmetries (Sy5) and their 6 modes of function (Sy6)
Elements or objects of the group	Altered scrutinies	LODs of fractal time
Invariant	Object identification	PS or PSI
Classification	Self-centered 3D spatiality	Self-centered events; gestalt “worlds”
Symmetry breaking as resetting	Not in CG, but implicit in the function of DQ	Enables SyTO to maintain PSI

See text for details.

**S4 in Now: One, Classification**. We consider how classification is founded on fractal time. First, we justify the use of fractal time. We then outline how fractal time can be modeled. Finally, we indicate how fractal time supports function in Now.

### Justification for the use of fractal time

First, fractal time is the proposed structure for temporal integration in PCM by encompassing the three nows that constitute the Now (Vrobel, 2011). These nows are primal impression, retention, and protention (i.e., the immediate present, past, and future). Second, we contend that AEEN SyTBM realizes the mapping from CG to Now. And in that regard, AEEN refers to self-organization involving CG. Self-organization implies the following: one, the non-stationary dynamics of structured flows on fractal manifolds (Pillai and Jirsa, 2017); two, that dynamic core theory proposes rapid, reciprocating, and repeated (re-entrant) neural interactions function as a scale-free dynamics of hierarchical self-similarity (Tononi and Edelman, 1998); and three, self-organized criticality leads to function in fractal space-time (Bak, 1997).

### How fractal time supports multimodal synchronic integration in Now

We now stipulate the properties of fractal time to show how it can support multimodal synchronic integration (Vrobel, 2007, 2011). Fractal time provides the grounding for the unified multi-level perspective for the observer-participant, i.e., circular causality involved in the perception of self in purposeful action. The multi-level perspectives consist of multiple levels of description that are defined in terms of a group-like structure characterized by the properties of closure, associativity, identity, and a unique inverse relationship (List, 2019). Circular causality is the *a priori* of all experience and knowledge in that the decision-making of the observer-participant creates the individual temporal observer perspective, a customized Now, a selection from many superposed states.

Fractal time supports the symintentry of self ~ other in terms of a scale-free dynamic of hierarchical self-similarity and dissimilarity. Fractal embedding creates three levels of embodiment (see Metzinger, 2007b; in Vrobel, 2011) that underlies the pre-reflective, corporeal relation to the world (see embodied hermeneusis below) as conceived by Merleau-Ponty (Brender, 2013).

Our Now is our only (pure) interface with the world. Fractal time encompasses the three “nows” that constitute the Now. Fractal time enables flow through time, simultaneously to travel back in time (retention) and project forwards in time (i.e., protention). Nesting Nows provide nested Nows with a context for the self-motivated experience. Contextualizing means nesting the past into the present Now and that into an anticipated future. The structuring of the self through fractal time underpins our sense of integrity and continuity and the depth of meaning in each moment of experience.

Fractal time provides for a temporal hierarchy, enabling extensive synchronicity. Synchronicity realized through nesting underpins precognition and our emotional sense of time that is essential for integrated, embodied, and phenomenal experience of Now. We contend that fractal time enables the expression of the subjective and objective capabilities of the symintentry operator (we further develop the subjective capacity in the section on Embodied hermeneusis below).

**S4 in Now: Two, Transformations**. Transformation is the second aspect of S4 that is realized through SyTO. By interacting with classification, SyTO realizes the group of symmetry transformations underlying the phenomenal self. These transformations underpin the invariance of the phenomenal self in the fractal time function in Now. SyTO enables the transformations of gestalts that are formulated in Now. That is, SyTO enables reconstructions of the form of PS as information processing. Symintentry provides the context for all transformations (automorphisms, autopoiesis) that enable the gestalt form of PS to become “other.”

**S4 in Now: Three, Invariance**. We have seen from studying the Erlangen program that particular categories, e.g., geometry (as considered by Klein), are determined through the study of invariants or structures preserved under symmetry transformations. Every particular (e.g., geometry), in its general concept and aim, is a theory of invariants with respect to a certain group, and the special nature of each depends upon the choice of this group (Schiemer, 2020).

We see that phenomenal self-identity is the invariant (PSI) that is central to transforming and transformations in Now. PS is maintained by symintentry functioning as PMIR governance over and selection (specification) of events produced by the perception of self in goal-directed action.

How can we further advance the notion that PS is maintained invariant in Now? Chester (2002) proposes that group logic formalizes how science (and we say the individual) can identify entities in the physical world in a consistent and repeatable manner. He claims that symmetry coincides with identification and identity. In that regard, we contend that phenomenal self-identity is the most singular and distinctive aspect of PS that must be maintained invariant in Now.

We will contend in Section 2 that PSI is maintained invariant through the SyTO reconciling extensive complementarities. The complementarities give PS meaning by remaining integral within the phenomenal experience and physical process Now. The integrity of PSI is an allostatic process.

**S4 in Now: Four, symmetry breaking**. We previously indicated in the properties of the DQ that DQ operations lead to a symmetry breaking or actually a symmetry resetting process that underpins intentionality. Through symmetry breaking, symintentry realizes transformations that enable PS to become all (any) “other” in each moment of self-reflective (transcendental Bitbol and Osnaghi, 2016) awareness.

### Overview: structure and function of Now in terms of S4

We have indicated the properties of each facet of S4. We specify how the facets realize the structure and function of Now (conscious processing) using AEEN SyTO functions (as PMIR) as a control parameter. SyTO generates and maintains PS invariant as PSI within and central to events in phenomenal (fractal) space-time, the Now.

## Section 2: the adaptation of Now as S4

**Overview:** In Section 1, we considered the emergence (AEEN) of symintentry and the generation of the structure of Now in terms of S4. In this Section, we consider AEEN symintentry through the adaptation of AEEN CG in generating S4. In particular, we explicate ASH by reconciling the role of symintentry in terms of Shaw (2001) ecological thesis on intentionality. We show that to resolve symmetry-breaking SyTO transformations, it is necessary to reclassify (recategorize) PS by enslaving extensive complementarities to generate gestalts that serve as optimal goal paths in Now.

Shaw contends that physical, biological, and psychological processes are all implicated in the function of intentionality in order to transact the business of survival in the ecosystem. Intentionality coordinates psychological experiences, physical processes, and biological acts. Psychological experience (PE) refers to the awareness or conscious perception of biological action and physical process. Physical process (PP) refers to all types of physical processes effecting change. Biological action (BA) provides context or rules governing adaptive function and thereby impacts both psychological experience and physical process.

We contend that the efficacy of symintentry stems from its transcendental capacity to actively link the demands of the knowing subject and the definition of its object (Bitbol et al., 2009). Symintentry can integrate Shaw's thesis because it enslaves complementarities (~), reconciling the subjective and objective aspects of relationships. The complementarities reflect the fact that Now function at metastability resolves the integration ~ segregation tendencies reconciling self ~ other.

Therefore, we see that symintentry is the ideal control parameter because it can reconcile PE ~ PP in Now. That is, symintentry reconciles and transcends the physical, biological, phenomenal, and psychological worlds by transforming the self into other Now. Consequently, we propose three levels of symintentry-based enslaving that reconcile PE ~ PP.

### Symintentry can support semiosis (BA ~ EH)

We first show how symintentry can support semiosis. The SyTO functions through rotation, translation, scaling, reflection, inversion, and extensive iteration operations to realize classification. The operations can realize gist ~ focus by moderating the interplay between time density and time length (see Vrobel, 2011).

Time density correlates with getting the gist, simultaneity, and maximal utilization of the nesting capacity of fractal time. Getting the gist also correlates with scaling up or emergent function. At the highest levels, these operations can account for the transcendental function alluded to previously (Bitbol et al., 2009). On the other hand, time length relates to focus on one or several LODs. Time length is a denesting phenomenon.

We propose that the symintentry function through gist ~ focus is analogous to the intentional faculty described in Husserl (1970) transcendental phenomenology. The gist ~ focus faculty of symintentry can resolve objective reference and interpretative sense (Hopp, 2008; Bar-Elli, 2013) or the particular way in which meaning can ensue.

The gist ~ focus function of symintentry resonates with our proposal that symintentry realizes intentionality by providing context for global transformations of PS. We contend that in AEEN, symintentry, as a control parameter, operates in the manner of biological action (BA) to realize the particular way meaning ensues through embodied hermeneusis (Halák, 2023).^10^

Overall, symintentry enslaves BA ~ EH to model and interpret psychological (phenomenal) experience (PE) ~ physical process (PP) in formulating goal paths as a gestalt.

### Symintentry controls communication

Symintentry function through fractal time realizes extensive synchronicity that, in Shaw (2001) terms, coordinates the intentional act and the intended referenced experience. Synchronicity provides the lawful basis for dynamically linking the “reference maker” and the “reference interpreter.” This connection guarantees that invariant information can exist across the shared social contexts through the sharing of intentional states (symintentry states) in fractal time, i.e., Now.

Overall, the global synthetic function of symintentry can realize the symbolic potential of embodied communication. We can define the structural basis for communication in terms of BA ~ EH structuring flows on the manifold of fractal time (as described by Jirsa, 2020) through which the embodied self communicates and becomes “other.”

We envisage that these dynamics describe communication in terms of the embodied PS being and becoming other, initially in the Now as a solitonic waveform. This waveform is then captured and simultaneously transformed into a standing wave and saved as an element of fractal time. The collection of standing waves realizes hermeneusis (EH) that is gained through the sharing of phenomenal experience by means of the physical process of fractal time. We see that the formation of standing waves is the basis for the information field that binds PSI in consciousness (field-of-consciousness; McFadden, 2020).

Overall, symintentry can control communication in Now by integrating symbolic potential (SB) with solitons (SO; SB ~ SO) as “symbolitons” or form ~ meaning constructions. Symbolitons are further instances of symintentry-mediated complementarities that can bridge the phenomenal ~ physical (subjective ~ objective) levels to realize global transformations of PS Now.

### Symintentry controls global complementarities

So far, we have specified how symintentry can control Now by generating complementarities that realize self ~ other semiotic relationships. These extensive complementarities, as hierarchies, consist of parallel streams governed by (PE ~ PP) ~ (BA ~ EH) ~ (SB ~ SO) ~ (~), where (~) refers to those multiple complementarities defined by Grossberg (2000, 2021). As he indicates, hierarchical interactions within each stream and parallel interactions between these streams can overcome the complementary deficiencies of each stream.

The key idea is that AEEN S4 (Sy5, Sy6) generates affordances for the adaptation of self ~ other coupling that we contend is through horizontal and vertically distributed metastable parallel streams functioning as a harmonic oscillator. The SyTO facilitates the resonation of this structure in concert with (PE ~ PP) ~ (BA ~ EH) ~ (SB ~ SO) ~ (~) to generate global gestalt patterns (Hunt and Schooler, 2019). Gestalts are conceived in terms of emergence, reification, invariance, and multistability (Lehar, 2003). Reification realizes harmonic resonance patterns as standing waves (Lehar, 2003) that we contend can underpin information processing in symbolic and solitonic modes (the basis for what we call “symbolitons”). These resonations capture events as fractal transformations of the phenomenal self as “future” becoming assimilated as “past” Now.

We see that symintentry resolves symmetry breaking in the most efficacious manner by structuring flows on the manifold of fractal time. Flows create coherent behavioral representations in the consciousness (Jirsa, 2020) that maintain (PSI) invariant despite symintentry transformations. Alternatively, Now realizes representations through which self (PSI) can become any “other” self that is chosen in each moment.

### AEEN S4 fulfills St5

We have shown how ASH is resolved through AEEN symintentry realizing St5 (see summary in Table 2).

**Table 2:** The resolution of ASH by correlating AEEN symintentry and St5.

**AEEN symintentry**	**Phenomenal invariants St5**
DQ linear algebra and matrix operations	Situated 3-Dimensional spatiality
Fractal time provides the manifold for Now.	Temporal integration
Now supports symintentry enslaving (PE ~ PP) ~ [(BA ~ EH) ~ (SB ~ SO)] ~ (~)	Multimodal synchronic integration
Symintentry is the control parameter governing S4	PMIR
S4 constructs and maintains PS invariant as PSI	Unique sense of “Selfhood”

**Figure 2:**
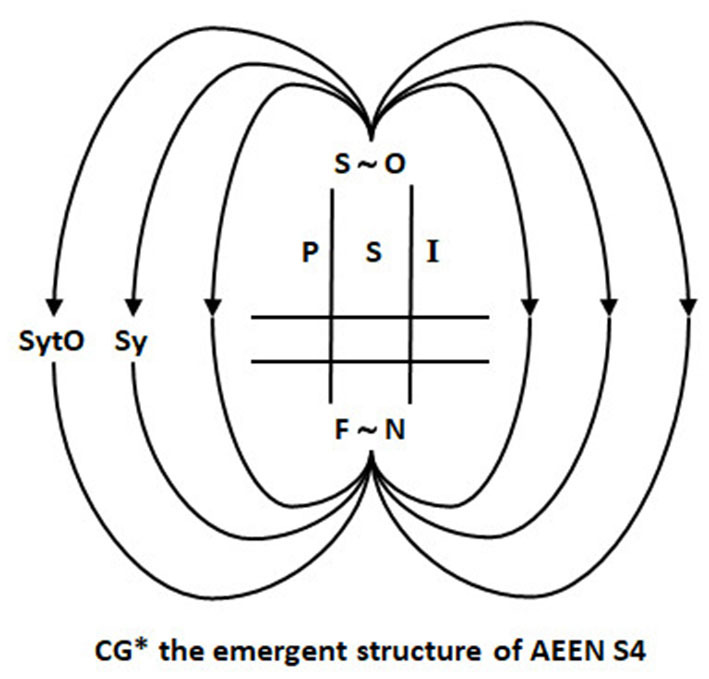
How the Ouroboros loop structure of Now realizes the conservation of PSI. The figure depicts the central core of Now as horizontal and vertically distributed, metastable, and parallel streams that adapt through resonance as a harmonic oscillator. This oscillator responds to symmetry breaking and projection of self as “other.” SyTO facilitates the creation of any or all potential futures through realizing the latent potential within the noumenal self (depicted as F ~ N) to become any or all “others” Now. The Ouroboros loop structure expedites “becoming” by the autocatalytic stimulation of symbols (Sy) and intentionality (In). Phenomenal self-identity (PSI) is conserved through symintentry operations.

**Figure 3:**
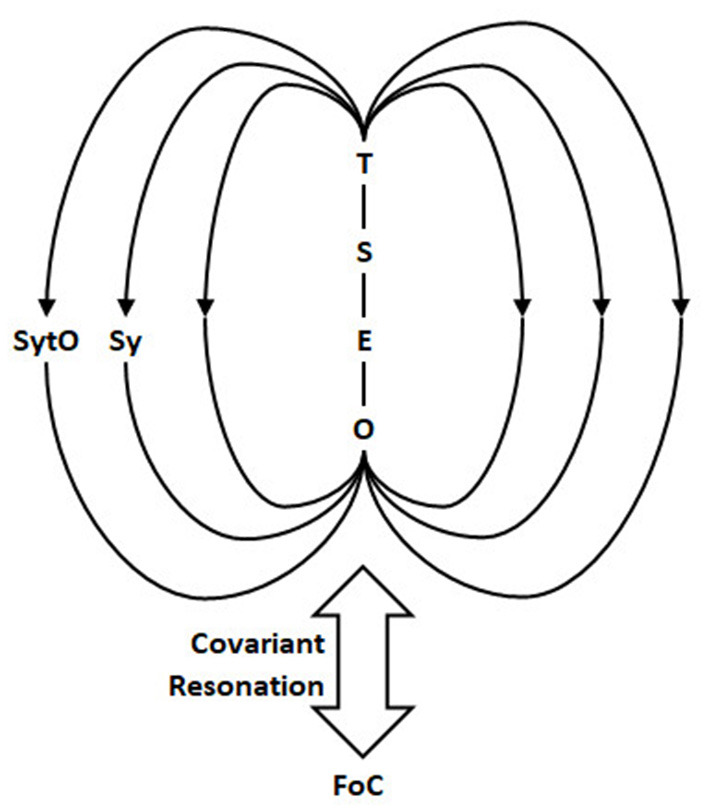
The Now as CG^*^ resonates with the field of consciousness (FoC). We propose that the conservation of PSI depends on the self-resonation of CG^*^ core structure transcendental-semiotic-epistemic-ontic (TSEO). SyTO “tunes” its core through the Ouroboros loop to resolve S ~ O Now. SyTO functions as an infinitesimal level (quantum) operator to synch the infinitely small perturbations to covary and become one with the field of consciousness (FoC).

Our findings are as follows:

First, 3D situated spatiality: We show by determining CG and realizing the mathematical properties of the nilpotent dual number, unit DQs can directly generate scaling operations necessary for a projective geometry. Second, CG develops under AEEN symintentry to realize fractal time, which is the manifold for temporal and multimodal synchronic integration. We call this integration S4. Furthermore, the emergence of S4 (Sy5, Sy6) forms CG^*^ through realizing affordances to form gestalts that resolve self ~ other Now. SyTO organizes the four-level structure of CG^*^ through self-referential Ouroboros loops. The loops underpin the symbolic and intentional role of embodied hermeneusis as symbolitons. We envisage that SyTO realizes self-reference and conserves PSI (see Figure 2) through the covariance of the Ouroboros loop within the field of consciousness (see Figure 3).

We see that the self-referential Ouroboros loop is key to the integrated function of Now. The loop realizes extensive adaptive resonance of S4 required to integrate past, present, and future into fractal time. The synchronicity is enhanced through symbolism and intentionality (symintentry). The loop structure creates a continuous feedback loop. So Now is not isolated but rather a dynamic temporal structure that incorporates a rich tapestry of temporal elements in conscious experience. These elements consist of recurrent patterns, thoughts, or experiences that repeat cyclically as individuals revisit certain thoughts or emotions. Furthermore, the loop connects the whole to its constituent parts, which fosters a sense of continuity and coherence in one's subjective reality. The loop also underpins regeneration and transformation as each cycle of experience brings fresh insights and adaptations that contribute to personal development. Finally, the loop suggests that we embody the paradoxical nature of all experience and communication that raises questions about infinity, self-reference, and the nature of reality.

## Concluding remarks

We have explored the intersection of neurophenomenology and transcendental structuralism (see Figure 1) to determine the *a priori* structure of (the preconditions for) phenomenal experience. We find that Now has organized into a highly embedded structure that, by symmetry breaking (projection), resolves future ~ noumena and how future (goal or other) can be realized through phenomenal self-realization (of noumenal self).

**Is symintentry the archetypical form of symmetry?** We explore the proposal that the structure of consciousness is founded on a more fundamental symmetry than St5. However, in the process of justifying the proposal, we instead found that symintentry is the appropriate form of symmetry. Consequently, we now claim that symintentry is not just a new form of symmetry but argue that it is the archetypical form.

Symintentry originates from and subsequently enables symmetry-based modeling to become the pervasive influence in the forebrain governing structure and function. Intentionality acknowledges human agency as fundamental to the production of symmetry. Symintentry uses complementarity to account for the existence of elements that may appear to be binary oppositions but co-exist in the brain and interact in producing perceptions of symmetry in Now moments.

We support our view of symintentry in three ways. First, we outline the properties of symintentry. Second, we show that symintentry can redefine intentionality. Third, we indicate the key role of symintentry in defining Now as the ideal, transcendental structure of the phenomenal mind. We stress that validation of these views requires extensive future research.

We now summarize the many remarkable properties of symintentry gleaned through our thought experiment. First, the properties gained through the DQ operator in CG (see Section 1). Second, iteration of the DQ forms fractal time that serves as the manifold for Now. We outlined the properties of fractal time and indicated its fraxel form that we are now modeling (see Appendix 4). Third, the function of the SyTO in S4 depends on rotation, translation, reflection, inversion, scaling, and extensive iteration. SyTO interactions with classification enable PE ~ PP to realize gist ~ focus and the particular way in which meaning can ensue.

Fourth, the uniqueness of symintentry as an operator stems from embodying the subjective and objective features of modeling. We see that these features are essential for adaptation (Shaw, 2001). Fifth, symintentry functions just as biological action to govern PE ~ PP by enslaving extensive complementarities. In turn, each of the complementarities governs others to generate avalanches of self-organization involving the nervous system functioning at criticality. We propose that symbolitons are one major example of complementarity involved in realizing form ~ meaning constructions of PS. Sixth, symintentry as the order parameter functions as PMIR in conserving PSI. Seventh, the symintentry function through the six modes of symmetry-based modeling (Mouchet, 2013) redefines intentionality. We will now further illustrate this point.

### Symintentry redefines intentionality

We indicated that symintentry has unique properties that enable it to transcend the characteristic features of intentionality, such as being representational, qualitative, perspectival, and subjective (Williford et al., 2018). We now outline how symintentry can redefine intentionality by realizing the subjective and objective aspects of modeling required to structure consciousness.

We indicated many ways through which symintentry models the subjective aspects of relationships. For example, symintentry engages the LODs of fractal time through gist ~ focus and objective reference ~ interpretative sense. Furthermore, symintentry provides the synchronicity and context essential for the sharing of intentional states by realizing how embodied hermeneusis can be expressed in terms of symbolitons.

Alternatively, in objective terms, we propose that the six modes of symmetry-based modeling (Mouchet, 2013) make symintentry functional (“Sy6”). Specifically, symintentry classifies (categorizes), builds representations, constrains and unifies interactions in models, extracts universal properties, bridges integrable and chaotic dynamics, and predicts selection rules.

We now indicate aspects of our research that support Sy6 in redefining intentionality. First, classification. We have seen that symintentry-based classification links the Erlangen Program and the function of Now as S4. In Now, classification is essential for the intentional generation of form-meaning constructions.

Second, symintentry builds representations of the universal “other” that maintains PSI. Third, symintentry constrains and unifies interactions in models. We see that symintentry functions through information control to define the role of intentionality in adapting to the ecosystem (Shaw, 2001).

Fourth, symintentry extracts universal properties. Symintentry governs universal transformations of PS through modeling and semiosis. By sharing intentional states in Now, PS can communicate with or model to become all (any) “other.”

Fifth, symintentry bridges integrable and chaotic dynamics. The role of the symintentry operator in information storage (e.g., in fraxels; see note on fractal time modeling in Appendix 4) is enabled by the symmetry and linearizing properties of DQ mathematics. Sixth, symintentry predicts selection rules. Symintentry realizes effectivity ~ affordances involved in selecting optimal gestalt goal paths in Now. An affordance is an opportunity for action. In terms of our thesis, S4 realizes the potentials within “things” that can be utilized in turn for action (actually enaction in terms of goal seeking). The affordances are realized through the tripartite Ouroboros loop. We contend that phenomenal self-modeling affordances enable the infinite universal adaptability of the phenomenal self to transform to (become) any and all “others” in all times, places, and coordinate systems.

In summary, we have indicated how symintentry enables some redefinition of intentionality. However, it remains an open research question as to the applicability of these six entities in Now.

### Symintentry ~ metastability characterizes the role of Now

We now consider several ways symintentry enables us to understand and thereby define the critical role of Now in brain function. Subsequently, we summarize the all-encompassing role of symintentry in coordinating the embedded structure of Now.

We begin by outlining three steps showing how the combination of symintentry and metastability enables the definition of Now. First, symmetry itself is defined as the properties of a system that remain unchanged after being subjected to transformations or operations. Second, symintentry realizes that the phenomenal self remains invariant despite the transformation through which the self becomes any “other.”

Third, from self-organization to metastability, symintentry ~ metastability governs the extensive synchronicity that defines macro-micro commutation in Now. How?

Metastability (see footnote 5) realizes that these integration ~ segregation properties of the system co-exist and can be resolved simultaneously. In other words, the fact that self (the integrated or macro properties) and other (the specified, micro, or goal-directed aspects) can be simultaneously codefined and realized defines Now.

### Symintentry explains how consciousness can model itself just by its structure

We see that symintentry facilitates and operates the two-way subjective ~ objective mirror that engages PS in projective transformations as event gestalt in Now. Symintentry through (PE ~ PP) ~ (BA ~ EH) ~ (SB ~ SO) ~ (~) enables PS to participate in an iterative transformational dialogue (representing representations) with the universal “other” that realizes goal paths as gestalt forms of PSI. By functioning in this manner, we contend that symintentry *is* (constitutes) the internal, intrinsic self-modeling relation that holds between the system and itself of necessity and invariantly (Williford, 2014). Symintentry describes how consciousness, as a physically realized system, can model itself (maintain PSI) just through its structure. We contend that being and becoming “other” Now symintentry realizes pre-reflective self-consciousness (Williford et al., 2022). This is aperspectival in being non-representable and fully embodied.

In essence, we are continually modeling ourselves in the pure simplicity of being. This suggests that SyTO can “tune” the resonant frequencies in the four levels to minimize time (energy expenditure) and maximize nuanced meaning as required to maintain PSI in Now. Tuning can realize four modes of phenomenal experiencing Now. Level 1: Being without thought. The mode of existing fully adapted as “one with the world” (Brender, 2013). Level 2: Validating the current hypothesis or model as correct and suitable. Level 3: Requires a change to the existing model, the “other.” Level 4: Paradigm shifts requiring extensive changes to the individual's view of the world.

### Symintentry determines the “form” of Now that makes PS manifest in Now

We have demonstrated how symintentry can maintain the “form” of PS invariant as PSI. We now indicate the ways in which symintentry makes that “form” manifest in Now.

First, the “forms” of PS are embodied form ~ meaning constructions recorded as gestalts through the function of symbolitons in fractal time. This is the “form” of information processing gained through symintentry, realizing universal transformations of PS into other through automorphisms (autopoiesis) Now. Second, “forms” are the pictures and memories that symintentry constructs in being and becoming other Now. Third, “form” refers to the extensive “binding” of St5 that is gained through symintentry enslaving (PE ~ PP) ~ [(BA ~ EH) ~ (SB ~ SO)] ~ (~) that grounds PS Now. Fourth, symintentry determines the “form” and information processing potential of fractal time modeled through the formation of Fraxels (see note in Appendix 4). Finally, we contend that the transcendental function of imagination is essential to integrate all “forms” into gestalts Now. Imagination is a product of the three-level Ouroboros loop function of SyTO. Furthermore, we propose that these loops are akin to the “strange loop” of Hofstadter (2007). The Ouroboros loop proposal characterizes Cassirer's profound realization of the utility of Group theory and symmetry as the transcendental function unifying self ~ other. Fundamentally, group symmetry, functioning as a general-purpose mechanism, unifies artistic ~ scientific approaches to phenomenal self-modeling. Cassirer's unification enables a deep understanding of the universality of structural relationships in a wide range of contexts.

These manifestations of symintentry realize how “form” governs our perception and cognition. Through AEEN SyTBM, the self can transform and become any other Now. Symintentry enables the self to actualize the physical process through phenomenal experience, as in the transformation PP ~ PE. This is achieved through the perception of self in purposeful action by eliciting the extensive complementarities we outlined to resolve each focus of awareness.

Symintentry epitomizes Varela's neurophenomenology program, reflecting embodiment (Castoriadis, 1980), autopoiesis (Varela, 1981), and embodied cognition (Kiverstein and Miller, 2015).

Our conception of Now is consistent with the development of self-evidencing by generating sufficiently thick or deep generative models to minimize the surprise (i.e., maximize model evidence) expected following an action (Friston, 2018). Thickness and depth underwrite inferences about the counterfactual consequences of an action.

Temporal thickness involves embodying models of the future. The Now is consistent with Merleau Ponty's notion of history as a sedimentation (Chouraqui, 2012) in which time accumulates thickening. The verticality of Now is in opposition to the horizontal ontology of time and represents transcendence itself. For Merleau Ponty, time is a vertical journey through the thickness of being. The thickness is the space of the complementary encounter between the human creative power and the sedimented determinations inherited from the past. In terms of SyTBM, AEEN S4 addresses that complementarity. AEEN S4 realizes self-expression in time (Buonomano, 2017) through the extended Now comprising ontology [Merleau Ponty being in the world (Bitbol, 2020)], epistemology (Cassirer, 1944), and semiosis (Parszutowicz, 2015). Symintentry enables this universal vertical journey through the thickness of being to becoming anyone Now (Frith and Frith, 2005).

## How can symintentry fit into PCM in terms of free energy minimization through active inference?

We contend that symintentry instantiates active inference and free energy minimization (Friston, 2003; Friston et al., 2006) to realize the optimal goal path selection of Now. The continuous modeling in the Now is not just limited to a single stream but to streams running in parallel. At key stages, they incorporate branching to some degree with possible alternative modeled action(s). Given this premise, each model stream includes a series of deterministic model elements (DQ-based). Each is assessed and assigned a probability of success (by some criteria) for each branch, culminating in pathways. One (or possibly more) of these pathways leads to a best-favorable or least-worst outcome.

Each model stream is continually reviewed and updated in the Now as new information becomes available, and the probable outcomes are adjusted through Bayesian statistics. In this way, we not only continually model our possible futures, but we weigh them statistically to aid decision-making on what actions to take to meet our desires/aims/goals—i.e., to realize symintentry.

Overall, the two mathematical systems combine the deterministic elements for a particular course(s) of action with stochastically assessed (i.e., via Bayesian updating) probabilities of meeting one's immediate or longer-term goals.

### Symintentry reinterprets Kant's remarkable idea regarding predictive processing

Our approach enables us to reinterpret Kant's remarkable idea that remains active in predictive processing (Swanson, 2016). In Table 3, we correlate the reinterpretations gained from our thesis with five core aspects of predictive processing predicated on Kant's thesis. We see that our explication of Kant's thesis complements the Projective Consciousness Model and its extension to phenomenal selfhood. AEEN SyTBM is crucial for how predictive processing can realize the construction of St5 Now. Therefore, the question arises: can AEEN SyTBM enable us to determine the law behind our own nature as we discover how we resolve ASH Now? We contend that a deeper understanding of symintentry is critical for this discovery.

**Table 3:** Correlation of Swanson's criteria with AEEN symintentry.

**Swanson's criteria**	**AEEN symmetry**
Five core aspects of predictive processing that were predicted by Kant	How the aspect is explicated by AEEN symintentry in Now
Objects conform to our cognition	AEEN symintentry governs semiotic Self ~ Other relationships in Now
Role of hyperpriors: Space and time constrain our perception of objects	The structure of PS is generated and maintained as gestalt events constructed in fractal space-time
Generative models or schemata mediate between images and concepts	SyTO enslaves extensive complementarities that prescribe gestalt goal paths
Cognitive-perceptual understanding proceeds through alternating iterative steps of analysis and synthesis	The symintentry operator functions through gist ~ focus to resolve the particular way in which meaning can ensue
Imagination is the key to the synthesis that underpins perception and understanding	We contend imagination is a product of the three-level Ouroboros loop function of SyTO

### We contend that symintentry is a least action principle

Symintentry has major selective advantages because it combines the properties of SyBM, the most powerful means of modeling in the physical objective world, with intentionality and the optimal means of modeling in the phenomenal world. We contend that symintentry realizes Occam's razor by functioning as a least action optimizing principle. Therefore, AEEN SyTBM facilitates the development of structure and function in Now by reducing complexity and the information processing requirements for the development of conscious processing.

We argue that symintentry is the archetypical form of symmetry. This proposal should stimulate future research on Kant's (and Cassirer's) paradigm shift to define the lawful basis for Now and how that can structure the phenomenal mind.

### The key implications and hypotheses arising from the AEEN symintentry hypothesis

We tried to validate ASH through a thought experiment that is essentially self-explicatory. That is, in order to validate the thought experiment, we used our own AEEN S4 in our Nows. We reconciled this study using our biosemiotic process that has developed (and continues to develop) through the emergence of inference and modeling. Therefore, our thesis is based on positing a wide-ranging number of hypotheses and attempting to resolve them. Ultimately, our thesis depends on the future validation of these hypotheses by scientific and artistic means. We have listed below (also in Table 4) the key hypotheses and sub-hypotheses that require further exposition and briefly indicate their relevance for the future integration of multiple disparate disciplines currently under the purview of cognitive science.

**Table 4:** The implications and hypotheses arising from the Kant-Cassirer thesis.

**Hypothesis**		**Outline of hypothesis**
H1	a	Kant's Copernican revolutionary idea can be fully explicated through Cassirer's conjecture.
	b	ASH: AEEN S4 (as symintentry-based modeling) realizes the *a priori* structure of phenomenal experience (Now), and that is equivalent to the structure of consciousness (St5)
H2	a	Group symmetry is the transcendental function that mediates the relationship between the phenomenal self and objects (more universally “other”).
	b	AEEN S4 realizes the role of the transcendental function in generating a highly embedded structure: symmetry breaking (projection) [(F ~ N)(S ~ O)[AEEN [S4 (Sy5. Sy6) [A^*^ [Gestalts]]]]].
	c	This structure facilitates and enriches the lived experience by realizing affordances, enabling infinite adaptability of self to transform to (become) any and all “others” in all times, places, and coordinate systems
H3	The structure emerges through AEEN group symmetry to function as a higher-level group extension of CG (“CG^*^”). The efficacy of CG^*^ for lived experience depends on:	
	a	Least action principle
	b	Free energy minimization
	c	Realizing invariant perception ~ action ~ goal directed coupling (Gibson)
	d	Consciousness can model itself just by its structure
	e	Now functions in a lawful manner in terms of Noether's theorem
	f	CG^*^ function can be modeled as a fractal that theoretically extends to quantum levels
	g	CG^*^ functions through a renormalization group (RNG) process
H4	Now adaptation secures agent ~ world coupling through function as a harmonic oscillator	
	a	Self ~ other transformations are mediated through SyTO engaging Sy5 (rotation, translation, scaling, reflection, and inversion) by horizontal and vertically distributed, metastable, and parallel streams
	b	SyTO unifies four levels of phenomenal experience: transcendental, semiotic, epistemic, and ontic to function as an Ouroboros loop
	c	SyTO functions as the control parameter that tunes the loop to realize four modes of phenomenal self-modeling
H5		AEEN S4 realizes Cassirer's conjecture that group symmetry functions as a general-purpose mechanism that unifies artistic ~ scientific approaches to phenomenal self-modeling
H6		The Occam's razor postulate is that AEEN S4 and Now enables the redefinition of “cognition,” “perception,” “intentionality,” “symbolism,” etc.
H7		The group-symmetry process realizes the field of consciousness
H8		The embedded process outlined is further embedded in biological process, biosemiotic process, and Bohm's thesis
H9		Now is consistent with the mediative “Now”
H10		Symintentry is the archetypical form of symmetry

**H1**: Kant is recognized as the father of cognitive science (Brook, 2019). By elucidating his revolutionary idea (Kant et al., 1996) using Cassirer's conjecture (Cassirer, 1944), we gain a fresh understanding of the *a priori* structure of the mind (Heis, 2014) and how that enables us to prescribe the structure of our world. Indeed, this is the world prescribed through F ~ N. By positing the most imaginative futures, we can best utilize the most exceptional time machine (Vrobel, 2007; Buonomano, 2017) in the universe to reinvent a fresh subjective ~ objective basis for the world.

**H2**: Group symmetry is the transcendental function that mediates the relationship between the phenomenal self and objects (more universally “other”). AEEN S4 realizes the role of the transcendental function in generating our posited highly embedded *a priori* structure that unifies phenomenal experience. For instance, through the schematic structure [Symmetry breaking (projection) [(F ~ N) (S ~ O)[AEEN [S4 (Sy5. Sy6) [A^*^ [Gestalts]]]]] and the mediation of the Ouroboros loop, we can literally come “face to face” and explore our noumenal self every time we enter Now.

**H3**: CG^*^ is the fully extensive structure that is group-based from top to bottom, from gestalts all the way down to quantum levels [“structure all the way down” (Ladyman and Ross, 2009)]. Our group-based *a priori* thesis is founded through quaternions, and DQ is expressed in terms of one of the most fundamental groups (Lie group). This is the same type of structure that has enabled mankind to “know” the physical and mental worlds through (PE ~ PP) ~ [(BA ~ EH) ~ (SB ~ SO)] ~ (~)). This is the formulation through which we can utilize the affordances generated through AEEN S4 that enables us to adapt, enact, resonate, embrace, and embody the world. H3 a-g are six implications of the formulation that enable us to derive the law that can elucidate the pivotal role of the phenomenal self-invariance in structuring Now.

**H4**: From exploring the generation and function of the Ouroboros loop, we can determine the very origins of symbolism [in form meaning constructions (Goldberg, 1997)] and intentionality. We propose that H4 b and c imply that we can tune into the inherent frequencies of the structure of Now by utilizing our own horizontal and vertically distributed, metastable, parallel streams—our own Ouroboros loop.

**H5**: Our study is not only a homage to Kant but also to Cassirer, who is arguably the most eclectic and visionary philosopher of the 20^th^ century (Cassirer, 1923, 1950). Cassirer embraced the vision that the disciplines of arts and sciences are one. He conjectured that group symmetry functions as a general-purpose mechanism. Group symmetry is the transcendental function (Lassègue, 2020) that enables us to know and become not just the objects in the world but to fully embrace semiosis in that we can become one with our world (Leroux, 2011; Parszutowicz, 2015).

**H6**: This is the Occam's razor postulate that implies that AEEN S4 emerging to CG^*^ is the foundation for Now and so will enable us to redefine many key aspects (if not all) of cognitive science.

**H7**: We contend that with AEEN S4 emerging to CG^*^, we can now discover how our phenomenal selves enable universal resonation with “others.” “Others” embraces us (self-revelation) and other people. Resonance stems from the Ouroboros loops through symbolism and intentionality—the pervasive influences in the cosmos (Shaw, 2001).

**H7** leads on to **H8**, where the Now we discover must be consistent with insight into Now gained through meditation, e.g., Tolle (2010).

**H9**: We propose that the embedded process AEEN S4-CG^*^ we outlined is further embedded in biological and biosemiotic processes (Friston, 2018) and Bohm's holonomic thesis (Norton and Smith, 2020).

Finally, we postulate **H10** that symintentry is the archetypical form of symmetry. We have presented many arguments to support that contention. However, ultimately, the validity of **H10** will depend on future research involving artistic and scientific exploration in the Now.

## Data availability statement

The original contributions presented in the study are included in the article/Supplementary material, further inquiries can be directed to the corresponding author.

## Author contributions

DR wrote the body of the paper and AS developed and wrote about the mathematics. All authors contributed to the article and approved the submitted version.

## Footnotes

^1^Our addition.

^2^The predictive processing paradigm provides the foundation for PCM.

^3^See our further rationalization of Cassirer's view below.

^4^The perspectives are views of the one adaptive process. We consider them separately just for exposition.

^5^Metastability reconciles complementarity, the tendency for functional brain components to couple (integrate) and also to express their intrinsic, independent behavior (segregate) simultaneously. Complementarity or synergy between contrary processes (denoted by ~) determines the universal patterns that characterize cognition and behavior in response to symmetry breaking. In this study we use the tilde (~) to indicate processes that proposedly function as complementarities.

^6^Symmetry has become a powerful theoretical tool to guide and at times dictate how we construct models and theories. In terms of symmetry-based modeling, symmetry may facilitate: (1) Building of linear algebraic representations; (2) Constraining and unifying interactions in models; (3) Extracting universal properties in non- linear dynamics; (4) Reducing the degrees of freedom and bridging integrable and chaotic dynamics; (5) Predicting selection rules; and (6) Classifying.

^7^In phenomenal experience.

^8^Cassirer's group could be named after Chester. However, because we extend Chester's group to encompass Cassirer's thesis, we name the group after Cassirer.

^9^Projective geometry is an essential feature in the field of consciousness. We propose that moment to moment space-time information storage is based upon a projective space oriented to the field of consciousness, that employs DQ projective transformations to make the necessary changes to the perceived or conceived world view, including its orientation and scale. Using this method would require much less effort and complexity (both mathematical and computational) in the conventional transformations between ever varying projective spaces.

^10^Fractal embedding creates three levels of embodiment.
