# Supplementary material for: Re-evaluating the structure of consciousness through the symintentry hypothesis
Source: Front Psychol. 2023 Nov 21;14:1005139. doi: 10.3389/fpsyg.2023.1005139 (PMC10712567; doi:10.3389/fpsyg.2023.1005139)
Supplement: Supplementary file 1 [file Data_Sheet_1.docx]

**Re-evaluating the Structure of Consciousness through the Symintentry Hypothesis.**

**Appendix 1: Precis on Self-organization:**

First, function at the network level([Pillai and Jirsa, 2017](#_ENREF_9)) ([Pillai and Jirsa, 2017](#_ENREF_9)) ([Jirsa, 2020](#_ENREF_8)): The brain self–organizes to conform to the nonstationary dynamics of structured flows on manifolds (SFMs) in Now. These properties are characterized by multistability, multiple time scales, intermittency, switching between attractor states, and noise-induced resonances.

Second, Dynamic core ([Tononi and Edelman, 1998](#_ENREF_11); [Edelman and Gally, 2001](#_ENREF_3)): dynamic core theory proposes that rapid, reciprocating and repeated (re-entrant) neural interactions function as a ‘‘scale-free dynamics” of hierarchical self-similarity. The dynamic core leads to successive discriminatory states, which entails information processing, and the emergence and supervenience of phenomenal experience.

Third, the global workspace ([Baars, 2007](#_ENREF_1)) is a dynamical, richly interactive, working memory which broadcasts messages to all participating systems. The global availability of information through the dynamical workspace is experienced as consciousness.

Fourth, after Grossberg ([Grossberg, 2000](#_ENREF_5)) ([Grossberg, 2021](#_ENREF_6)) neurological function depends on complementarity between parallel streams. Complementarity creates coherent behavioural representations that support unitary conscious experiences. Hierarchical interactions within each stream and parallel interactions between streams overcome the complementary deficiencies of each stream.

Fifth, self-organized criticality ([Bak, 1997](#_ENREF_2)) ([Werner, 2007](#_ENREF_12)) through self-organization to criticality CG-symmetry can function in fractal space-time.

Finally, we have indicated the important role of metastability in Coordination Dynamics previously (See footnote 5, Page 3 in the Main Text).

**Appendix 2: Dual Quaternion function.**

A general DQ is an 8-tuple which can be written as:

$\text{σ}\text{=}\left( \text{p}_{\text{s}}\text{+}\text{p}_{\text{x}}\text{i}\text{+}\text{p}_{\text{y}}\text{j}\text{+}\text{p}_{\text{z}}\text{k} \right)\text{+}\text{ε}\left( \text{q}_{\text{s}}\text{+}\text{q}_{\text{x}}\text{i}\text{+}\text{q}_{\text{y}}\text{j}\text{+}\text{q}_{\text{z}}\text{k} \right)$ and its conjugate is defined as:

$\sigma^{*}\text{=}\left( \text{p}_{\text{s}}-\text{p}_{\text{x}}\text{i}-\text{p}_{\text{y}}\text{j}-\text{p}_{\text{z}}\text{k} \right)\text{+ε}\left( \text{q}_{\text{s}}-\text{q}_{\text{x}}\text{i}-\text{q}_{\text{y}}\text{j}-\text{q}_{\text{z}}\text{k} \right)$

where the p terms represent a general quaternion and the q terms associated with the dual number (ε) represent a second independent general quaternion. For both p and q terms, the subscript “s” means a scalar/pseudoscalar term, while the x,y,z subscripts relate to the orthonormal x,y,z axes with the emboldened **i**,**j**,**k** represent unit quaternion “vectors” (strictly speaking these are actually bivectors), whose rotation axes are aligned with the directions of the x,y,z axes, respectively.

Aside from the scalar terms, the unit quaternion vectors are non-commutative and follow the rules that **ij**=**k**=-**ji**, **jk**=**i**=**-kj**, **ki**=**j**=**-ik** and **i**^2^ =**j**^2^=**k**^2^=**ijk**= -1. The Dual Number is nilpotent, where ε ≠ 0, yet ε^n^=0, where n≠1.

A unit DQ $\left( \hat{\sigma} \right)$ is defined as $\hat{\sigma}\hat{\sigma}^{*}=1$, where $\hat{\sigma}^{*}$is the quaternion conjugate and it can be shown that for this product to equal unity, then:

$\left( p_{s}^{2}+p_{x}^{2}+p_{y}^{2}+p_{z}^{2} \right)=1$and that $\left( p_{s}q_{s}+p_{x}q_{x}+p_{y}q_{y}+p_{z}q_{z} \right)=0$

and to satisfy this, the unit DQ has to take the form $\hat{\sigma}\text{=}\left( \text{1}\text{+}\text{0}\text{i}\text{+}\text{0}\text{j}\text{+}\text{0}\text{k} \right)\text{+}\text{ε}\left( \text{0}\text{+}\text{q}_{\text{x}}\text{i}\text{+}\text{q}_{\text{y}}\text{j}\text{+}\text{q}_{\text{z}}\text{k} \right)$ or more simply $\hat{\sigma}\text{=1+ε}\left( \text{q}_{\text{x}}\text{i }\text{+ }\text{q}_{\text{y}}\text{j }\text{+}\text{ q}_{\text{z}}\text{k} \right)$ and thus the unit conjugate is

$\hat{\sigma}\text{*=1}-\text{ε}\left( \text{q}_{\text{x}}\text{i }\text{+ }\text{q}_{\text{y}}\text{j}\text{+}\text{q}_{\text{z}}\text{k} \right)$.

It can be seen that the dual scalar term (q_s_) is zero and in fact if it is made non-zero, then errors will occur in the mathematical operation of combining rotations and translations. On the surface, this restriction appears to prevent the dual scalar from being non-zero and hence prevent the DQ from holding Galilean space-time coordinates.

However, it turns out that there is a simple mathematical process which means that Galilean coordinates can in principle be stored in a DQ in memory and subsequently be transformed into two components, namely geometric and temporal. But we first examine raising a DQ to a power. Consider a DQ of the form:

$\sigma\text{=1+ε}\left( q_{s}+\text{q}_{\text{x}}\text{i }\text{+ }\text{q}_{\text{y}}\text{j }\text{+}\text{ q}_{\text{z}}\text{k} \right)$ raised to the power n.

It can be shown using a series expansion that $\sigma^{n}$ scales the DQ linearly, due to the nilpotent number $\text{ε}$ properties:

$\sigma^{n}=\left( 1+\varepsilon\left( q_{s}+q_{x}\boldsymbol{i}+\text{ }q_{y}\boldsymbol{j}+q_{z}\boldsymbol{k} \right) \right)^{n}=1+n\varepsilon\left( q_{s}+q_{x}\boldsymbol{i}+\text{ }q_{y}\boldsymbol{j}+q_{z}\boldsymbol{k} \right)$

Using this property coupled with the conjugate, it can then be shown that:

$\begin{matrix} \left( \sigma^{1/2}\left( \sigma^{{-1}/2} \right)^{*} \right)=1+\varepsilon\left( q_{x}\boldsymbol{i}+\text{ }q_{y}\boldsymbol{j}+q_{z}\boldsymbol{k} \right)=\hat{\sigma} & The unit DQ \left( geometric component \right) \\ \left( \left( \sigma^{1/2} \right)^{*}\sigma^{1/2} \right)=1+\varepsilon\left( q_{s} \right)=\sigma_{t} & The temporal DQ component \end{matrix}$

And the quaternion product of these components: $\hat{\sigma}\sigma_{t}=\sigma$

We next introduce two other defined DQ conjugates ([Jia, 2013](#_ENREF_7)), the first is the dual conjugate that has the form $\mathrm{DQ}^{\bullet}\text{=}\left( \text{p}_{\text{s}}+\text{p}_{\text{x}}\text{i}+\text{p}_{\text{y}}\text{j}+\text{p}_{\text{z}}\text{k} \right)-\text{ε}\left( \text{q}_{\text{s}}+\text{q}_{\text{x}}\text{i}+\text{q}_{\text{y}}\text{j}+\text{q}_{\text{z}}\text{k} \right)$ and the second has a composite form: $\left( \mathrm{DQ}^{*} \right)^{\bullet}=\mathrm{DQ}^{◊}\text{=}\left( \text{p}_{\text{s}}-\text{p}_{\text{x}}\text{i}-\text{p}_{\text{y}}\text{j}-\text{p}_{\text{z}}\text{k} \right)+\text{ε}\left( {-\text{q}}_{\text{s}}+\text{q}_{\text{x}}\text{i}+\text{q}_{\text{y}}\text{j}+\text{q}_{\text{z}}\text{k} \right)$. This latter conjugate $\left( \mathrm{DQ}^{◊} \right)$ is widely used in the DQ transformations involving rotation then translation (and vice versa), as used in the “sandwich” function^[[1]](#footnote-1)^. Typically the rotation and translation transformations are associatively adjoined prior to their action on the initial unit DQ. As mentioned above this process precludes the use of the dual scalar.

In other words, DQs in this form can be stored as numerous sets of values, each one representing a point in Galilean space-time, each set representing an object relative to the “observer’s” local moving Cartan frame at each instant of the Now. Considering conceptual visualization, it is posited that sets of these DQs are selected and recalled, split into the two channels, the geometric component transformed (rotated, translated, projected (see Appendix 3) and/or reflected). We posit that this all takes place in each Now in a “timeless” mode (i.e. $q_{s}=0$) and then the time-step determined and joined back to the data. This implies that the time-step does not have to be current, it could be that or the past or the projected future, but saved with the current Now’s information (see Appendix 4 on the posited formation of temporal fractals).

**Appendix 3: Dual Quaternion projection and perspective**

An important feature of the DQ mathematics is that it can readily and straightforwardly deal with a dynamic geometric projection onto a part-spherical surface, i.e. the retina, as required for perception. This is achieved by projecting through the DQ’s origin, which acts a focal point and is also effectively the “observer’s origin of self”. Now in the unit DQ mathematics, this is not carried out by matrices, but via a simplified algorithm which makes use of the dual number’s nilpotency. It can be shown that for a unit DQ, projection of a point onto a part-spherical surface, centred at the DQ’s origin is

$\sigma^{{-r}/R}=\left( 1+\varepsilon\left( q_{x}\boldsymbol{i}+ q_{y}\boldsymbol{j}+q_{z}\boldsymbol{k} \right) \right)^{{-r}/R}=1-\frac{r}{R}\varepsilon\left( \text{q}_{\text{x}}\text{i }\text{+ }\text{q}_{\text{y}}\text{j }\text{+}\text{ q}_{\text{z}}\text{k} \right)$,

where r is the eye’s radius (which is negated as the image is inverted) and R is the distance from the origin to a point on the object – this is a scaled inversion. The DQ mathematics can further deal simply with the projection of a rotating object moving along some 3D path relative to the observer (who is also moving), by combining the DQ algorithms of rotation, translation, scaling, reflection and inversion. For example, let a point in 3-space be: $\sigma_{1}=1+\varepsilon\left( ix_{1}+jy_{1}+kz_{1} \right)$ where $\left( x_{1},y_{1},z_{1} \right)$ are the coordinates relative to the observer’s origin, with a radial distance of $R_{1}=\left\| \sigma_{1} \right\|$; then the observed projection (p_1_) will be:

$p_{1}=\left( 1+\varepsilon\left( ix_{1}+jy_{1}+kz_{1} \right) \right)^{-r/{R_{1}}}=1-\varepsilon\frac{r}{R_{1}}\left( ix_{1}+jy_{1}+kz_{1} \right)$.

If the observer moves position, then $\sigma_{2}=1+\varepsilon\left( ix_{2}+jy_{2}+kz_{2} \right)$ where $\left( x_{2},y_{2},z_{2} \right)$ are coordinates of the point on the object in its new position relative to the observer’s origin, with a new radial distance $R_{2}=\left\| \sigma_{2} \right\|$. The new observed projection of that point (p_2_) will be:

$p_{2}=\left( 1+\varepsilon\left( ix_{2}+jy_{2}+kz_{2} \right) \right)^{-r/{R_{2}}}=1-\varepsilon\frac{r}{R_{2}}\left( ix_{2}+jy_{2}+kz_{2} \right)$. It can thus be seen that this is straightforward linear mathematics, but with the significant advantage of allowing DQ rotation and translation of the point (in Euclidean space) relative to its former position, prior to updating the projection. Then by considering the surfaces of each object in 3-space to consist of myriads of points, each of which is moving relative to the observer, this mathematics allows continual update of the projection. The important aspect is that this projection method will form an accurate perspective on a spherical surface (the retina), as opposed to perspective on a planar surface. We contend that this method is comparable with the PCM model, but with the advantage of being founded on a single mathematical structure, i.e. the DQs.

Another key feature of this DQ projection method is that it has the potential to be used in reverse (driven by memory or imagination), in other words to generate conceptual images, similar to the “Lived Space” in Figure 4 in the PCM ([Williford et al., 2018](#_ENREF_13)). Given this proposal there is a very simple transformation using the same DQ mathematics of the projection, but transforming from Self to Other. For example, if say you conceive a specific object, together with the Other, you can by this means approximately transform your conception of the object from a different perspective, as if you had become the Other. This all works by first transforming the origin from the Self to the Other just prior to instigating the virtual projection and then finally transforming the origin back to the Self. This would mean that you can not only become the Other conceptually, but you can also transform perception of external objects as if you are “observing” them through the Other’s senses. The capacity of symintentry to transform Self and transform Self into any Other could be the source of control over personal and inter - subjective behaviours ([Rudrauf et al., 2022](#_ENREF_10)). It can also underlie the theory of Mind ([Frith and Frith, 2005](#_ENREF_4)). We can use the same mechanism we use for ourself recursively to make inferences about other’s states and predict their actions and beliefs ([Rudrauf et al., 2022](#_ENREF_10)).

**Appendix 4: Further notes on modelling fractal time**

In order to explore the potential of the DQ mathematical framework proposed, we carried out some initial investigative work modelling Fractal Time. This was based on the concept of a 3D Cantor type of fractal, generated by an iterated function system (IFS), using unit DQs. The IFS generates a fractal that in principle could store and massively compress information as a discrete sequential time-series. DQ iteration generates a highly compressed temporal fractal form with self-similar cubic fractal cells (“fraxels”), which can be in a space-filling manner. We posit that each of these fraxels could hold a snapshot version of the Now. The DQ mathematics driving and supporting the process ensures linearity of compression in an effective and efficient manner, while generating an accurate information framework that we posit could form a temporal fractal.

**Appendix 5: Symmetry breaking and the DQ**

One of the key features required for intentionality is the process of “symmetry breaking”. We contend that symmetry breaking, which implies a discontinuous jump from one state (A) to another (B) can be considered in a different way in this context. We argue that instead of just using some form of mathematical single step function (such as the Heaviside step function), that a two-stage process that we call “resetting” is more appropriate. Thus, instead of $A⟼B$, we define resetting as $A⟼N\mapsto B$, where N is neutral or an intermediate non-state. In order to achieve this in the DQ mathematics, we again make use of raising the DQ to a power n (as outlined in Appendixes 2 and 3):

$\sigma_{A}^{n}=\left( 1+\varepsilon\left( q_{xA}\boldsymbol{i} + q_{yA}\boldsymbol{j} +q_{zA}\boldsymbol{k} \right) \right)^{n}=1+n\varepsilon\left( q_{xA}\boldsymbol{i} + q_{yA}\boldsymbol{j} +q_{zA}\boldsymbol{k} \right)$

then letting n tend to zero: $\lim_{n\to0} \sigma_{a}^{n}=1+0\times\varepsilon\left( q_{xA}\boldsymbol{i} + q_{yA}\boldsymbol{j} +q_{zA}\boldsymbol{k} \right)=1$

(As expected by raising any number to the power of zero). Importantly, this means that the DQ dual vectors are zero (i.e. at the DQ’s 3D origin) and thus the DQ is in a non-state. Hence mathematically the symmetry breaking (or resetting) could be written along the lines of $\sigma_{A}^{n\left( t \right)}\sigma_{B}^{m\left( t \right)}$ where $n\left( t \right), m\left( t \right)$ are some functions of time t. This would allow all types of state change ranging from (i) state A ramping down to neutral, then state B ramping up, to (ii) an abrupt state change from state A to state B (i.e. the non-state time tends to zero).

Baars, B.J. (2007). The global workspace theory of consciousness. *The Blackwell companion to consciousness***,** 236-246.

Bak, P. (1997). *How nature works.* Oxford university press Oxford.

Edelman, G.M., and Gally, J.A. (2001). Degeneracy and complexity in biological systems. *Proceedings of the National Academy of Sciences* 98(24)**,** 13763-13768.

Frith, C., and Frith, U. (2005). Theory of mind. *Current biology* 15(17)**,** R644-R645.

Grossberg, S. (2000). The complementary brain: Unifying brain dynamics and modularity. *Trends in cognitive sciences* 4(6)**,** 233-246.

Grossberg, S. (2021). Conscious Mind, Resonant Brain: How Each Brain Makes a Mind. Oxford University Press.

Jia, Y.-B. (2013). Dual quaternions. *Iowa State University: Ames, IA, USA*.

Jirsa, V. (2020). "Structured Flows on Manifolds as guiding concepts in brain science," in *Selbstorganisation–ein Paradigma für die Humanwissenschaften*. Springer), 89-102.

Pillai, A.S., and Jirsa, V.K. (2017). Symmetry breaking in space-time hierarchies shapes brain dynamics and behavior. *Neuron* 94(5)**,** 1010-1026.

Rudrauf, D., Sergeant-Perthuis, G., Belli, O., Tisserand, Y., and Serugendo, G.D.M. (2022). Modeling the subjective perspective of consciousness and its role in the control of behaviours. *Journal of Theoretical Biology* 534**,** 110957.

Tononi, G., and Edelman, G.M. (1998). Consciousness and complexity. *science* 282(5395)**,** 1846-1851.

Werner, G. (2007). Metastability, criticality and phase transitions in brain and its models. *Biosystems* 90(2)**,** 496-508.

Williford, K., Bennequin, D., Friston, K., and Rudrauf, D. (2018). The projective consciousness model and phenomenal selfhood. *Frontiers in Psychology* 9**,** 2571.

1. The “sandwich function” is the transformation of a unit DQ by conjugation. Conventionally for a translation followed by a rotation, this takes the form $\left( RT \right)\hat{\sigma}\left( RT \right)^{◊}=RT\sigma T^{◊}R^{◊}$, where R and T are half angles and half translations, respectively and the operator ◊ is as defined in the text. The braces around the terms R and T indicate adjoining the terms associatively. [↑](#footnote-ref-1)
